# Supplementary material for: Content-rich biological network constructed by mining PubMed abstracts
Source: BMC Bioinformatics. 2004 Oct 8;5:147. doi: 10.1186/1471-2105-5-147 (PMC528731; doi:10.1186/1471-2105-5-147)
Supplement: Additional File 2 — The original results of the above study (non-essential files are deleted to keep the file size under the limit set by BMC bioinformatics). [file 1471-2105-5-147-S2.bz2 › chilibotAdditionalFile2/dip05/611ID10580496E10994/html/ATPE_ATPB.html]

 


 **ATPE** and **ATPB** 
  
Found 26 abstracts in PubMed, retrieved 05.  
 

 What does Google say? 
 PDF only 
| .edu only 

---

**Interactive relationship** (e.g. stimulation, inhibition, etc)

**Non-interactive relationship** (e.g. studied together, co-existance, homology, etc.)

- The ninth KpnI fragment about 4.0 kb of broad bean chloroplast DNA contains the entire chloroplast  **atpE**  gene and  **atpB**  gene.  Ref: 12168017 Sheng Wu Hua Xue Yu Sheng Wu Wu Li Xue Bao Shanghai < MedlineTA>Sheng Wu Hua Xue Yu Sheng Wu Wu Li Xue Bao Shanghai ,
- The genes for the five subunits were present in the order atpI subunit I  [ **ATPB** ] ,  **atpB**  subunit a,  **atpE**  subunit c, atpX subunit b, and atpF subunit b.  Ref: 9818357 Arch Microbiol, 1998
- The genes were organized in an operon with the gene order atpEBFHAGDC ; i.e., the order of  **atpE**  and  **atpB**  is reversed with respect to the more typical bacterial organization.  Ref: 10940012 J Bacteriol, 2000
- No genes for photosynthetic functions have been found, except for sequences encoding six subunits of the ATP synthase atpA,  **atpB** ,  **atpE** , atpF, atpH, and atpI.  Ref: 12111556 Mol Genet Genomics, 2002
- Eight genes atpI,  **atpB** ,  **atpE**  1,  **atpE**  2,  **atpE**  3, atpF, atpH, and atpA upstream of and contiguous with the previously described genes atpG, atpD, and atpC were cloned from chromosomal DNA of Acetobacterium woodii.  Ref: 10567365 J Biol Chem, 1999
